# Supplementary material for: What does it take to learn the rules of RNA base pairing? A lot less than you may think
Source: bioRxiv. 2025 Aug 2:2025.07.31.668042. Preprint. [Version 1] doi: 10.1101/2025.07.31.668042 (PMC12324431; doi:10.1101/2025.07.31.668042)
Supplement: Supplement 1 [file media-1.gz › supplemental_material/src/rscape_v2.6.0.tar.gz!/rscape_v2.6.0/lib/infernal/Manuscripts/banded-cyk/tbl-transitions.doc]

| dist | tied | dist | grp | node | next |  | Dirichlet α parameters | | | | | | | | | | | | | |
| --- | --- | --- | --- | --- | --- | --- | --- | --- | --- | --- | --- | --- | --- | --- | --- | --- | --- | --- | --- | --- |
| # | to | cts | cts | state | node |  | \|α\| | *t* | |  | *t* |  | *t* |  | *t* |  | *t* |  | *t* |  |
| 1 |  | 6 | 11 | MATP_MP | BIF |  | 0.5509 | | IL | 0.1229 | IR | 0.0001 | B | 0.8770 |  |  |  |  |  |  |
| 2 |  | 7023 | 7119 | MATP_MP | MATP |  | 7.2986 | | IL | 0.0023 | IR | 0.0024 | MP | 0.9816 | ML | 0.0056 | MR | 0.0046 | D | 0.0035 |
| 3 |  | 1600 | 1830 | MATP_MP | MATL |  | 1.5914 | | IL | 0.0179 | IR | 0.0155 | ML | 0.9200 | D | 0.0466 |  |  |  |  |
| 4 |  | 145 | 195 | MATP_MP | MATR |  | 1.9038 | | IL | 0.0173 | IR | 0.0073 | MR | 0.8903 | D | 0.0852 |  |  |  |  |
| 5 | 1 | 2 | 11 | MATP_MP | END |  | 0.5509 | | IL | 0.1229 | IR | 0.0001 | E | 0.8770 |  |  |  |  |  |  |
| 6* |  | 1 | 2 | MATP_ML | BIF |  | 3.0000 | | IL | 0.3333 | IR | 0.3333 | B | 0.3333 |  |  |  |  |  |  |
| 7 |  | 577 | 577 | MATP_ML | MATP |  | 0.6941 | | IL | 0.0131 | IR | 0.0103 | MP | 0.4032 | ML | 0.4983 | MR | 0.0115 | D | 0.0636 |
| 8 |  | 133 | 133 | MATP_ML | MATL |  | 0.9316 | | IL | 0.0739 | IR | 0.0651 | ML | 0.7038 | D | 0.1571 |  |  |  |  |
| 9 |  | 15 | 15 | MATP_ML | MATR |  | 0.3272 | | IL | 0.1884 | IR | 0.0432 | MR | 0.4082 | D | 0.3602 |  |  |  |  |
| 10* | 6 | 1 | 2 | MATP_ML | END |  | 3.0000 | | IL | 0.3333 | IR | 0.3333 | E | 0.3333 |  |  |  |  |  |  |
| 11* |  | 1 | 2 | MATP_MR | BIF |  | 3.0000 | | IL | 0.3333 | IR | 0.3333 | B | 0.3333 |  |  |  |  |  |  |
| 12 |  | 531 | 531 | MATP_MR | MATP |  | 0.7987 | | IL | 0.0079 | IR | 0.0190 | MP | 0.3241 | ML | 0.0193 | MR | 0.5631 | D | 0.0666 |
| 13 |  | 151 | 151 | MATP_MR | MATL |  | 0.6933 | | IL | 0.0357 | IR | 0.0699 | ML | 0.3066 | D | 0.5879 |  |  |  |  |
| 14 |  | 15 | 15 | MATP_MR | MATR |  | 0.3574 | | IL | 0.0582 | IR | 0.0002 | MR | 0.7629 | D | 0.1787 |  |  |  |  |
| 15* | 11 | 1 | 2 | MATP_MR | END |  | 3.0000 | | IL | 0.3333 | IR | 0.3333 | E | 0.3333 |  |  |  |  |  |  |
| 16* |  | 0 | 2 | MATP_D | BIF |  | 3.0000 | | IL | 0.3333 | IR | 0.3333 | B | 0.3333 |  |  |  |  |  |  |
| 17 |  | 575 | 575 | MATP_D | MATP |  | 0.5450 | | IL | 0.0019 | IR | 0.0047 | MP | 0.0857 | ML | 0.0534 | MR | 0.0528 | D | 0.8015 |
| 18 |  | 149 | 149 | MATP_D | MATL |  | 0.5831 | | IL | 0.0421 | IR | 0.0526 | ML | 0.2080 | D | 0.6973 |  |  |  |  |
| 19 |  | 14 | 14 | MATP_D | MATR |  | 0.1164 | | IL | 0.0001 | IR | 0.0001 | MR | 0.2439 | D | 0.7559 |  |  |  |  |
| 20* | 16 | 2 | 2 | MATP_D | END |  | 3.0000 | | IL | 0.3333 | IR | 0.3333 | E | 0.3333 |  |  |  |  |  |  |
| 21 |  | 2 | 4 | MATP_IL | BIF |  | 1.4397 | | IL | 0.6553 | IR | 0.0445 | B | 0.3002 |  |  |  |  |  |  |
| 22 |  | 121 | 126 | MATP_IL | MATP |  | 0.9402 | | IL | 0.1673 | IR | 0.1394 | MP | 0.5904 | ML | 0.0443 | MR | 0.0259 | D | 0.0327 |
| 23 |  | 114 | 119 | MATP_IL | MATL |  | 0.8046 | | IL | 0.3108 | IR | 0.1936 | ML | 0.4610 | D | 0.0346 |  |  |  |  |
| 24 |  | 14 | 15 | MATP_IL | MATR |  | 1.0926 | | IL | 0.1419 | IR | 0.0501 | MR | 0.6538 | D | 0.1541 |  |  |  |  |
| 25 | 21 | 2 | 4 | MATP_IL | END |  | 1.4397 | | IL | 0.6553 | IR | 0.0445 | E | 0.3002 |  |  |  |  |  |  |
| 26 |  | 1 | 31 | MATP_IR | BIF |  | 0.9361 | | IR | 0.2827 | B | 0.7173 |  |  |  |  |  |  |  |  |
| 27 |  | 145 | 227 | MATP_IR | MATP |  | 1.5494 | | IR | 0.1884 | MP | 0.7090 | ML | 0.0165 | MR | 0.0588 | D | 0.0273 |  |  |
| 28 |  | 129 | 701 | MATP_IR | MATL |  | 1.6332 | | IR | 0.3681 | ML | 0.5752 | D | 0.0566 |  |  |  |  |  |  |
| 29 |  | 8 | 160 | MATP_IR | MATR |  | 1.2428 | | IR | 0.2633 | MR | 0.6809 | D | 0.0558 |  |  |  |  |  |  |
| 30 | 26 | 0 | 31 | MATP_IR | END |  | 0.9361 | | IR | 0.2827 | E | 0.7173 |  |  |  |  |  |  |  |  |
| 31 |  | 108 | 1130 | MATL_ML | BIF |  | 1.2298 | | IL | 0.0078 | B | 0.9922 |  |  |  |  |  |  |  |  |
| 32 |  | 420 | 1319 | MATL_ML | MATP |  | 2.4162 | | IL | 0.0132 | MP | 0.9520 | ML | 0.0150 | MR | 0.0129 | D | 0.0070 |  |  |
| 33 |  | 19013 | 19371 | MATL_ML | MATL |  | 1.8632 | | IL | 0.0082 | ML | 0.9711 | D | 0.0207 |  |  |  |  |  |  |
| 34 |  | 859 | 6692 | MATL_ML | MATR |  | 72.1283 | | IL | 0.0058 | MR | 0.9755 | D | 0.0187 |  |  |  |  |  |  |
| 35 | 31 | 801 | 1130 | MATL_ML | END |  | 1.2298 | | IL | 0.0078 | E | 0.9922 |  |  |  |  |  |  |  |  |
| 36 |  | 28 | 172 | MATL_D | BIF |  | 6.8008 | | IL | 0.0029 | B | 0.9971 |  |  |  |  |  |  |  |  |
| 37 |  | 103 | 103 | MATL_D | MATP |  | 0.7288 | | IL | 0.0321 | MP | 0.5730 | ML | 0.0536 | MR | 0.1654 | D | 0.1758 |  |  |
| 38 |  | 3152 | 3152 | MATL_D | MATL |  | 0.4101 | | IL | 0.0138 | ML | 0.3105 | D | 0.6756 |  |  |  |  |  |  |
| 39 |  | 154 | 154 | MATL_D | MATR |  | 0.6736 | | IL | 0.0203 | MR | 0.6014 | D | 0.3782 |  |  |  |  |  |  |
| 40 | 36 | 144 | 172 | MATL_D | END |  | 6.8008 | | IL | 0.0029 | E | 0.9971 |  |  |  |  |  |  |  |  |
| 41 | 26 | 13 | 31 | MATL_IL | BIF |  | 0.9361 | | IL | 0.2827 | B | 0.7173 |  |  |  |  |  |  |  |  |
| 42 | 27 | 35 | 227 | MATL_IL | MATP |  | 1.5494 | | IL | 0.1884 | MP | 0.7090 | ML | 0.0588 | MR | 0.0165 | D | 0.0273 |  |  |
| 43 | 28 | 549 | 701 | MATL_IL | MATL |  | 1.6332 | | IL | 0.3681 | ML | 0.5752 | D | 0.0566 |  |  |  |  |  |  |
| 44 | 29 | 45 | 160 | MATL_IL | MATR |  | 1.2428 | | IL | 0.2633 | MR | 0.6809 | D | 0.0558 |  |  |  |  |  |  |
| 45 | 26 | 0 | 31 | MATL_IL | END |  | 0.9361 | | IL | 0.2827 | E | 0.7173 |  |  |  |  |  |  |  |  |
| 46 | 31 | 206 | 1130 | MATR_MR | BIF |  | 1.2298 | | IR | 0.0078 | B | 0.9922 |  |  |  |  |  |  |  |  |
| 47 | 32 | 848 | 1319 | MATR_MR | MATP |  | 2.4162 | | IR | 0.0132 | MP | 0.9520 | ML | 0.0150 | MR | 0.0129 | D | 0.0070 |  |  |
| 48 | 34 | 5833 | 6692 | MATR_MR | MATR |  | 2.1283 | | IR | 0.0058 | MR | 0.9755 | D | 0.0187 |  |  |  |  |  |  |
| 49 |  | 39 | 39 | MATR_D | BIF |  | 0.4664 | | IR | 0.0463 | B | 0.9537 |  |  |  |  |  |  |  |  |
| 50 |  | 176 | 176 | MATR_D | MATP |  | 0.8689 | | IR | 0.0245 | MP | 0.6126 | ML | 0.1269 | MR | 0.0471 | D | 0.1890 |  |  |
| 51 |  | 771 | 771 | MATR_D | MATR |  | 0.4869 | | IR | 0.0119 | MR | 0.3373 | D | 0.6507 |  |  |  |  |  |  |
| 52 | 26 | 15 | 31 | MATR_IR | BIF |  | 0.9361 | | IR | 0.2827 | B | 0.7173 |  |  |  |  |  |  |  |  |
| 53 | 27 | 39 | 227 | MATR_IR | MATP |  | 1.5494 | | IR | 0.1884 | MP | 0.7090 | ML | 0.0165 | MR | 0.0588 | D | 0.0273 |  |  |
| 54 | 29 | 107 | 160 | MATR_IR | MATR |  | 1.2428 | | IR | 0.2633 | MR | 0.6809 | D | 0.0558 |  |  |  |  |  |  |
| 55 |  | 338 | 338 | BEGL_S | MATP |  | 5.0422 | | MP | 0.9579 | ML | 0.0121 | MR | 0.0183 | D | 0.0117 |  |  |  |  |
| 56 | 31 | 15 | 1130 | BEGR_S | BIF |  | 1.2298 | | IL | 0.0078 | B | 0.9922 |  |  |  |  |  |  |  |  |
| 57 | 32 | 51 | 1319 | BEGR_S | MATP |  | 2.4162 | | IL | 0.0132 | MP | 0.9520 | ML | 0.0150 | MR | 0.0129 | D | 0.0070 |  |  |
| 58 | 33 | 358 | 19371 | BEGR_S | MATL |  | 1.8632 | | IL | 0.0082 | ML | 0.9711 | D | 0.0207 |  |  |  |  |  |  |
| 59 | 26 | 2 | 31 | BEGR_IL | BIF |  | 0.9361 | | IL | 0.2827 | B | 0.7173 |  |  |  |  |  |  |  |  |
| 60 | 27 | 3 | 227 | BEGR_IL | MATP |  | 1.5494 | | IL | 0.1884 | MP | 0.7090 | ML | 0.0588 | MR | 0.0165 | D | 0.0273 |  |  |
| 61 | 28 | 19 | 701 | BEGR_IL | MATL |  | 1.6332 | | IL | 0.3681 | ML | 0.5752 | D | 0.0566 |  |  |  |  |  |  |
| 62 | 1 | 3 | 11 | ROOT_S | BIF |  | 0.5509 | | IL | 0.1229 | IR | 0.0001 | B | 0.8770 |  |  |  |  |  |  |
| 63 | 2 | 96 | 7119 | ROOT_S | MATP |  | 7.2986 | | IL | 0.0023 | IR | 0.0024 | MP | 0.9816 | ML | 0.0056 | MR | 0.0046 | D | 0.0035 |
| 64 | 3 | 230 | 1830 | ROOT_S | MATL |  | 1.5914 | | IL | 0.0179 | IR | 0.0155 | ML | 0.9200 | D | 0.0466 |  |  |  |  |
| 65 | 4 | 50 | 195 | ROOT_S | MATR |  | 1.9038 | | IL | 0.0173 | IR | 0.0073 | MR | 0.8903 | D | 0.0852 |  |  |  |  |
| 66 | 21 | 0 | 4 | ROOT_IL | BIF |  | 1.4397 | | IL | 0.6553 | IR | 0.0445 | B | 0.3002 |  |  |  |  |  |  |
| 67 | 22 | 5 | 126 | ROOT_IL | MATP |  | 0.9402 | | IL | 0.1673 | IR | 0.1394 | MP | 0.5904 | ML | 0.0443 | MR | 0.0259 | D | 0.0327 |
| 68 | 23 | 5 | 119 | ROOT_IL | MATL |  | 0.8046 | | IL | 0.3108 | IR | 0.1936 | ML | 0.4610 | D | 0.0346 |  |  |  |  |
| 69 | 24 | 1 | 15 | ROOT_IL | MATR |  | 1.0926 | | IL | 0.1419 | IR | 0.0501 | MR | 0.6538 | D | 0.1541 |  |  |  |  |
| 70 | 26 | 0 | 31 | ROOT_IR | BIF |  | 0.9361 | | IR | 0.2827 | B | 0.7173 |  |  |  |  |  |  |  |  |
| 71 | 27 | 5 | 227 | ROOT_IR | MATP |  | 1.5494 | | IR | 0.1884 | MP | 0.7090 | ML | 0.0165 | MR | 0.0588 | D | 0.0273 |  |  |
| 72 | 28 | 4 | 701 | ROOT_IR | MATL |  | 1.6332 | | IR | 0.3681 | ML | 0.5752 | D | 0.0566 |  |  |  |  |  |  |
| 73 | 29 | 0 | 160 | ROOT_IR | MATR |  | 1.2428 | | IR | 0.2633 | MR | 0.6809 | D | 0.0558 |  |  |  |  |  |  |

Table 1: **Dirichlet priors for transitions.** “dist #”= index for the 73 different types of transition distributions in CMs; asterisks mark six distributions which had very few observed counts even after tying into groups, and for which we assigned a uniform plus-one Laplace prior. “tied to”= if an index is shown in this column, this distribution was estimated in a group (pooling observed counts) with the indicated distribution. “dist counts”= total counts observed for this distribution in the training data, before pooling into groups. “grp counts”= total counts for a group of one or more pooled distributions; this is the size of the training data sets for 36 different single-component Dirichlet priors. “node state”= unique CM state type the transition is from. “next node”= the node type that the transitions are going to. The codes for node types and state types in a CM are more fully explained in [16].
